# Association between dietary protein intake and prostate cancer risk: evidence from a meta-analysis

**DOI:** 10.1186/s12957-018-1452-0

**Published:** 2018-07-24

**Authors:** Ye Mao, Yan Tie, Jing Du

**Affiliations:** Cancer Center, West China Hospital, West China Medical School Sichuan University, No. 37, Guoxue Alley, Chengdu, 610041 Sichuan Province China

**Keywords:** Dietary, Protein intake, Prostate cancer, Meta-analysis

## Abstract

**Background:**

Many studies were conducted to explore the relationship between dietary protein intake and risk of prostate cancer, obtaining inconsistent results. Therefore, this study aims to comprehensively explore the predicted role of dietary protein intake for risk of prostate cancer.

**Methods:**

Databases of Web of Knowledge, PubMed, Chinese National Knowledge Infrastructure (CNKI), and Wan Fang Med Online were searched up to August 30, 2017. Eligible studies were included based on our definite inclusion criteria. Summarized relative risk (RR) and corresponding 95% confidence interval (CI) were pooled with a random effects model. Sensitive analysis and publication bias were performed.

**Results:**

At the end, a total of 12 articles comprising 13,483 prostate cancer cases and 286,245 participants were included. The summary RR and 95%CI of the highest protein intake compared to those with the lowest protein intake on prostate cancer risk were 0.993 (95%CI = 0.930–1.061), with no between-study heterogeneity found (*I*^2^ = 0.0%, *P* = 0.656). Moreover, the association was not significant on prostate cancer risk with animal protein intake [RR = 1.001, 95%CI = 0.917–1.092] or vegetable protein intake [RR = 0.986, 95%CI = 0.904–1.076]. The results were not changed when we conducted subgroup analysis by study design, cancer type, or geographic locations. We did not detect any publication bias using Egger’s test (*P* = 0.296) and funnel plot.

**Conclusion:**

Our study concluded that protein intake may be not associated on prostate cancer.

## Background

Prostate cancer is one of the most common cancer among men, and nearly a million new cases are diagnosed worldwide [1]. In total, the incidence rate of prostate cancer in western countries is higher than that in other countries [2, 3]. The reason for this status may be the differences in dietary intake [3, 4]. In western countries, they usually eat foods rich in calories, saturated fats, as well as animal protein, and so on. However, lower intake of fruits, vegetables, and whole grains lead to diet imbalance. Therefore, these western diets are not only related to prevalence of obesity [5] but also can directly change the known parameters to promote the growth of prostate cancer [6].

Protein is macromolecules made of amino acids and have basic functions in all known biologic processes. As we all know, protein contains 22 known amino acids. Of these, 9 essential amino acids could not be synthesized in the body [7]. Therefore, humans must eat some levels of foods which are rich in protein, to obtain the essential amino acid that is required for new protein synthesis. The protein usually comes from animal meats, plants such as soy, and dairy products [7]. Many publications were performed to assess the association about prostate cancer with high-protein intake. However, the effect on prostate cancer from different studies remains to be controversial. To address this question, we sought to perform this comprehensive meta-analysis to reflect the current totality of evidence on the subject.

## Methods

### Literature search

Articles were searched from the electronic searches of Web of Knowledge, PubMed, Chinese National Knowledge Infrastructure (CNKI), and Wan Fang Med Online, with the strategy of ‘protein’ OR ‘nutrition’ OR ‘diet’ AND ‘prostate cancer’ OR ‘prostate oncology’ as recent as August 30, 2017. Moreover, the bibliographies of searched publications were cross-referenced in order to identify additional articles.

### Inclusion and exclusion criteria

The inclusion criteria in this meta-analysis were (1) observational studies or experimental studies; (2) studies assessing the association about prostate cancer with protein intake; (3) the relative risk (RR) with the corresponding 95% confidence interval (CI) in the relation was available, or could be calculated basing on relevant data; (4) reporting the studies on humans; and (5) studies published in English language or Chinese language.

By contrast, the studies were excluded if they (1) reported on animal studies or cell studies; (2) were reviews, letter to the editors, or comments; and (3) contained insufficient data for statistical analysis.

### Data extraction

The following required data were abstracted according to a predefined standardized form: the first author’s last name; publication years; prostate cancer type; protein (total protein, animal protein, or vegetable protein); region for the study; study type; mean age or age range; follow-up duration; cases and participants; RR with 95%CI for the association between dietary protein intake and risk of prostate cancer; and adjustment for covariates. Two independent individuals extracted the data and the disagreements were resolved by a third reviewer.

### Statistical analysis

RR with the corresponding 95%CI was combined to calculate the summary results [8]. A chi-square test *I*^2^ statistic was used to assess the heterogeneity [9], and *I*^2^ < 25%, *I*^2^ = 25–50%, *I*^2^ > 50% suggested low, moderate, and high heterogeneity [10]. All the analysis used random effects model as pooled results. Sensitivity analysis was performed to find if some single study affected the overall results or not. Egger’s test [11] and Begg’s funnel plots [12] were utilized to examine the publication bias. Stata 12.0 software (STATA, College Station, TX, USA) was used to carry out the statistical analyses. *P* < 0.05 defined statistical significance.

## Results

### Search results and characteristics of studies

**Fig. 1:**
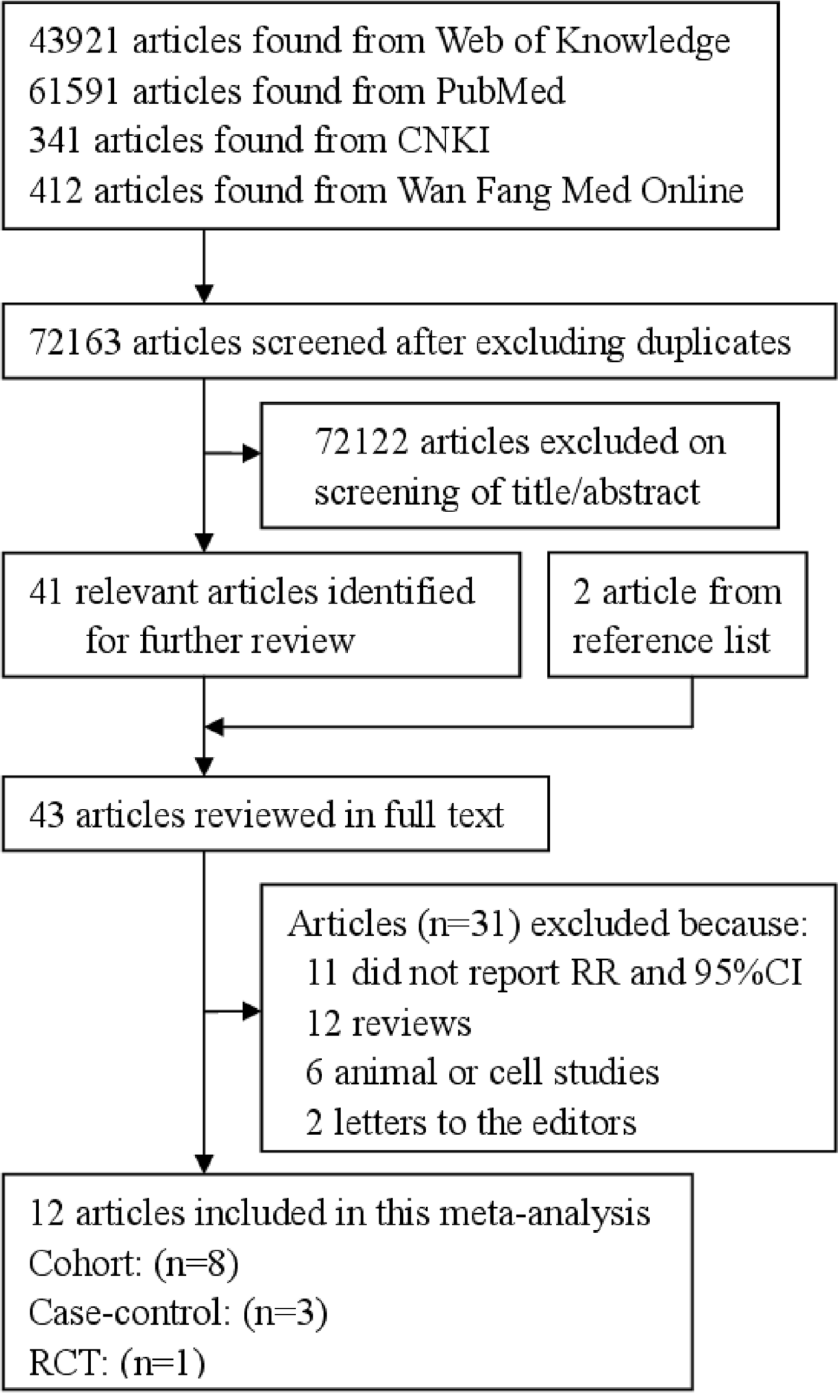
Study selection process for this meta-analysis

Figure 1 shows the flow diagram. The initial screening identified 43,921 articles from Web of Knowledge, 61,591 articles from PubMed, 341 articles from Chinese National Knowledge Infrastructure (CNKI), and 412 articles from Wan Fang Med Online. After the duplicated publications from different databases were excluded and the title and abstract reviewed, 41 articles were further reviewed for full text. There are 2 additional articles that were searched from the references of reviewed articles. Eleven articles that did not obtain RR and 95%CI, 12 review articles, 6 animal or cell articles, and 2 letters to the editors were further excluded. Therefore, 12 articles [13–24] were left for this study. Eight studies were cohort design, 5 studies were case-control design, and the remaining 1 study was RCTs. Six studies came from Europe, 5 from America, and 1 from Asia. All of the suitable studies included 13,483 prostate cancer cases and 286,245 participants. The characteristics of the included studies are summarized in Table 1.

**Table 1:** Characteristics of the included studies about the association of dietary protein intake on prostate cancer risk

Study, year	Design	Age	Participants; cases	Country	Follow-up duration	Protein type	Category	RR (95%CI)	Adjustment
Allen NE, 2008	Cohort	60.3 ± 6.7	142,251; 2727	European countries	8.7	Total protein	Total protein	Total protein	Adjusted for education, marital status, height, weight, and energy intake
						Animal	80 g/day	1	
						Vegetable	90 g/day	0.93 (0.82–1.05)	
							98 g/day	0.90 (0.78–1.04)	
							105 g/day	1.00 (0.85–1.18)	
							121 g/day	1.17 (0.96–1.44)	
							Animal	Animal	
							47 g/day	1	
							59 g/day	0.88 (0.77–1.00)	
							64 g/day	0.84 (0.73–0.97)	
							69 g/day	0.99 (0.86–1.15)	
							80 g/day	0.97 (0.81–1.15)	
							Vegetable	Vegetable	
							29 g/day	1	
							33 g/day	1.09 (0.96–1.22)	
							36 g/day	1.04 (0.91–1.19)	
							38 g/day	1.08 (0.93–1.26)	
							47 g/day	1.01 (0.82–1.23)	
Andersson SO, 1996	Case-control	70.7 ± 5.9	1056; 524	Sweden	NA	Total protein	Quartile 1	1	Adjusted for age and energy
							Quartile 2	0.90 (0.63–1.28)	
							Quartile 3	1.10 (0.78–1.55)	
							Quartile 4	1.07 (0.76–1.50)	
Berndt SI, 2002	Cohort	46–92	454; 69	USA	3.5	Animal	36.8 g/day	1	Adjusted for age and energy
							48.0 g/day	0.94 (0.49–1.82)	
							62.8 g/day	1.06 (0.56–2.01)	
Chan JM, 2000	Cohort	50–69	27,062; 184	Finland	8	Total protein	82 g/day	1	Adjusted for supplementation group, education, and quintiles of age, body mass index, energy, and number of years as a smoker
							94 g/day	0.9 (0.6–1.3)	
							102 g/day	0.6 (0.4–1.0)	
							107 g/day	0.8 (0.5–1.3)	
							117 g/day	1.0 (0.7–1.6)	
Deneo-Pellegrini H, 1999	Case-control	40–89	408; 175	Uruguay	NA	Total protein	Quartile 1	1	Adjusted for age, residence, urban/rural status, education, family history of prostate cancer, body mass index and total energy intake
							Quartile 2	1.1 (0.6–1.0)	
							Quartile 3	1.7 (0.9–1.3)	
							Quartile 4	1.0 (0.6–1.8)	
Kristal AR, 2010	RCT	63.6 ± 5.6	9559; 1703	USA and Canada	NA	Total protein	Quartile 1	1	Adjusted for age, race/ethnicity, treatment arm, and body mass index
							Quartile 2	1.00 (0.86–1.17)	
							Quartile 3	0.96 (0.82–1.12)	
							Quartile 4	0.93 (0.79–1.08)	
Lane JA, 2017	Cohort	63.0 ± 6.5	5245; 1717	UK	13.3	Total protein	Quartile 1	1	Adjusted for age, BMI, socioeconomic, smoking and marital status, diabetes and energy intake
							Quartile 2	1.00 (0.82–1.23)	
							Quartile 3	1.16 (0.95–1.42)	
							Quartile 4	1.02 (0.83–1.25)	
							Quartile 5	1.03 (0.83–1.29)	
Mills PK, 1989	Cohort	74	14,000; 180	USA	6	Vegetable	< 1 serving/week	1	Adjusted for age
							1–4 serving/week	0.83 (0.59–1.16)	
							> 4 serving/week	0.67 (0.40–1.12)	
Schuurman AG, 1999	Cohort	63.9 ± 3.8	58,279; 642	Netherlands	6.3	Total protein	Total protein	Total protein	Adjusted for age, family history of prostate cancer, socioeconomic status and total energy intake
						Animal	62 g/day	1	
						Vegetable	69 g/day	1.04 (0.76–1.43)	
							75 g/day	1.12 (0.82–1.53)	
							81 g/day	1.35 (0.98–1.84)	
							90 g/day	1.10 (0.81–1.51)	
							Animal	Animal	
							34 g/day	1	
							42 g/day	1.29 (0.92–1.81)	
							47 g/day	1.16 (0.80–1.68)	
							53 g/day	1.52 (1.01–2.30)	
							64 g/day	1.32 (0.76–2.29)	
							Vegetable	Vegetable	
							22 g/day	1	
							25 g/day	0.86 (0.63–1.17)	
							27 g/day	1.00 (0.74–1.36)	
							30 g/day	0.83 (0.61–1.14)	
							35 g/day	0.90 (0.66–1.23)	
Severson, RK 1989	Cohort	NA	7999; 174	Japanese	18	Total protein	0–74.9 g	1	Adjusted for age
							75.0–99.9 g	1.54 (1.07–2.22)	
							100.0+ g	1.13 (0.76–1.67)	
Smit E, 2007	Cohort	45–64	9777; 167	Puerto Rico	12	Total protein	Total protein	Total protein	Adjusted for age, education, body mass index, living, physical activity, smoking and residual energy intake. Calories not adjusted for energy intake
						Animal	≤ 61 g	1	
						Vegetable	62–82 g	0.94 (0.60–1.48)	
							83–103 g	1.02 (0.64–1.63)	
							≥ 104 g	1.32 (0.81–2.17)	
							Animal	Animal	
							≤ 13 g	1	
							14–23 g	0.78 (0.49–1.24)	
							24–40 g	0.91 (0.55–1.51)	
							≥ 41 g	1.01 (0.52–1.96)	
							Vegetable	Vegetable	
							≤ 16 g	1	
							17–23 g	1.27 (0.82–1.96)	
							24–31 g	1.07 (0.66–1.75)	
							≥ 32 g	1.19 (0.66–2.13)	
Tsilidis KK, 2013	Case-control	67.3 ± 5.4	10,155; 5221	European countries	NA	Total protein	Total protein	Total protein	Adjusted for continuous age at blood draw, continuous body mass index and energy consumption
						Animal	Tertile 1	1	
						Vegetable	Tertile 2	0.96 (0.87–1.06)	
							Tertile 3	0.95 (0.86–1.05)	
							Animal	Animal	
							Tertile 1	1	
							Tertile 2	1.05 (0.95–1.16)	
							Tertile 3	1.00 (0.90–1.11)	
							Vegetable	Vegetable	
							Tertile 1	1	
							Tertile 2	0.98 (0.89–1.09)	
							Tertile 3	1.00 (0.90–1.11)	

*Abbreviation*: *RR* relative risk, *CI* confidence intervals, *RCT* randomized controlled trial, *NA* not available

### Meta-analysis

**Fig. 2:**
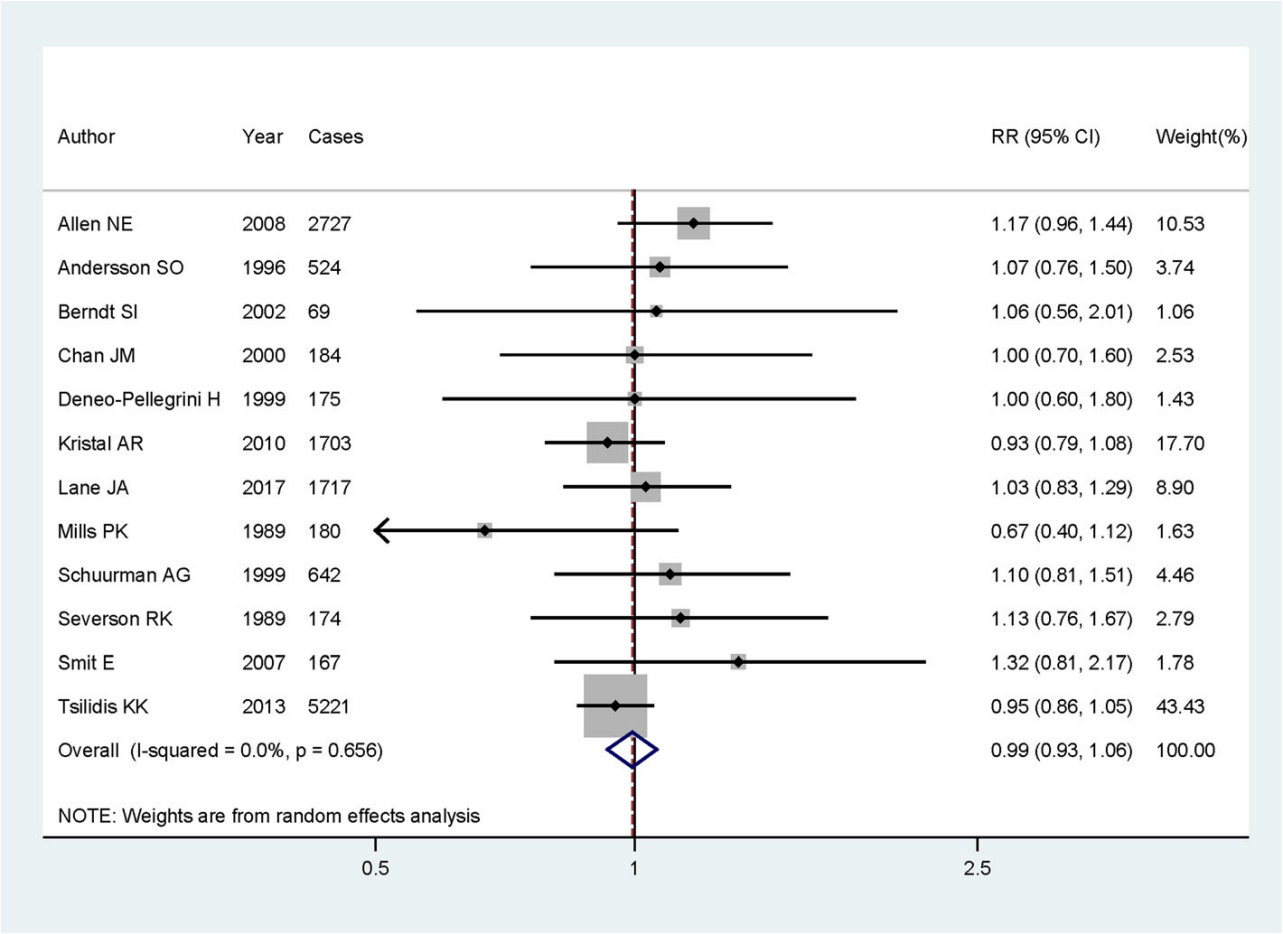
Forest plot for assessment of association between dietary protein intake and risk of prostate cancer

In the overall analysis, the summary RR and 95%CI of the highest protein intake compared to those with the lowest protein intake on prostate cancer risk were 0.993 (95%CI = 0.930–1.061), with no between-study heterogeneity found (*I*^2^ = 0.0%, *P* = 0.656) (Fig. 2).

In the stratified analysis by protein type, the association was significant on prostate cancer risk neither in animal protein intake [RR = 1.001, 95%CI = 0.917–1.092] nor in vegetable protein intake [RR = 0.986, 95%CI = 0.904–1.076]. There is no significant association found either in cohort studies [RR = 1.080, 95%CI = 0.964–1.209] or in case-control studies [RR = 0.960, 95%CI = 0.874–1.055]. The summary RRs (95%CI) of the highest protein intake compared to the lowest protein intake were 1.263 (95%CI = 0.953–1.674) on prostate cancer localized-stage disease risk and 0.973 (95%CI = 0.745–1.272) on prostate cancer advanced-stage disease. The results in the subgroup analysis by geographic locations were not changed. Detailed results are showed in Table 2.

**Table 2:** Summary RR and 95%CI of the association between dietary protein intake and prostate cancer risk

Subgroups	Number of studies	RR	95%CI	*P* for trend	Heterogeneity test	
					*P*	*I*^2^ (%)
Overall	12	0.993	0.930–1.061	0.841	0.656	0.0
Protein type						
Animal protein	5	1.001	0.917–1.092	0.988	0.891	0.0
Vegetable protein	5	0.986	0.904–1.076	0.753	0.556	0.0
Study design						
Cohort	8	1.080	0.964–1.209	0.184	0.670	0.0
Case-control	3	0.960	0.874–1.055	0.399	0.797	0.0
RCT	1	–	–	–	–	–
Cancer type						
Localized-stage disease	2	1.263	0.953–1.674	0.103	0.508	0.0
Advanced-stage disease	3	0.973	0.745–1.272	0.843	0.703	0.0
Geographic locations						
Europe	6	1.005	0.931–1.085	0.899	0.566	0.0
America	5	0.943	0.824–1.080	0.397	0.450	0.0
Asia	1	–	–	–	–	–

*RR* relative risk, *CI* confidence interval, *RCT* randomized controlled trial

**Fig. 3:**
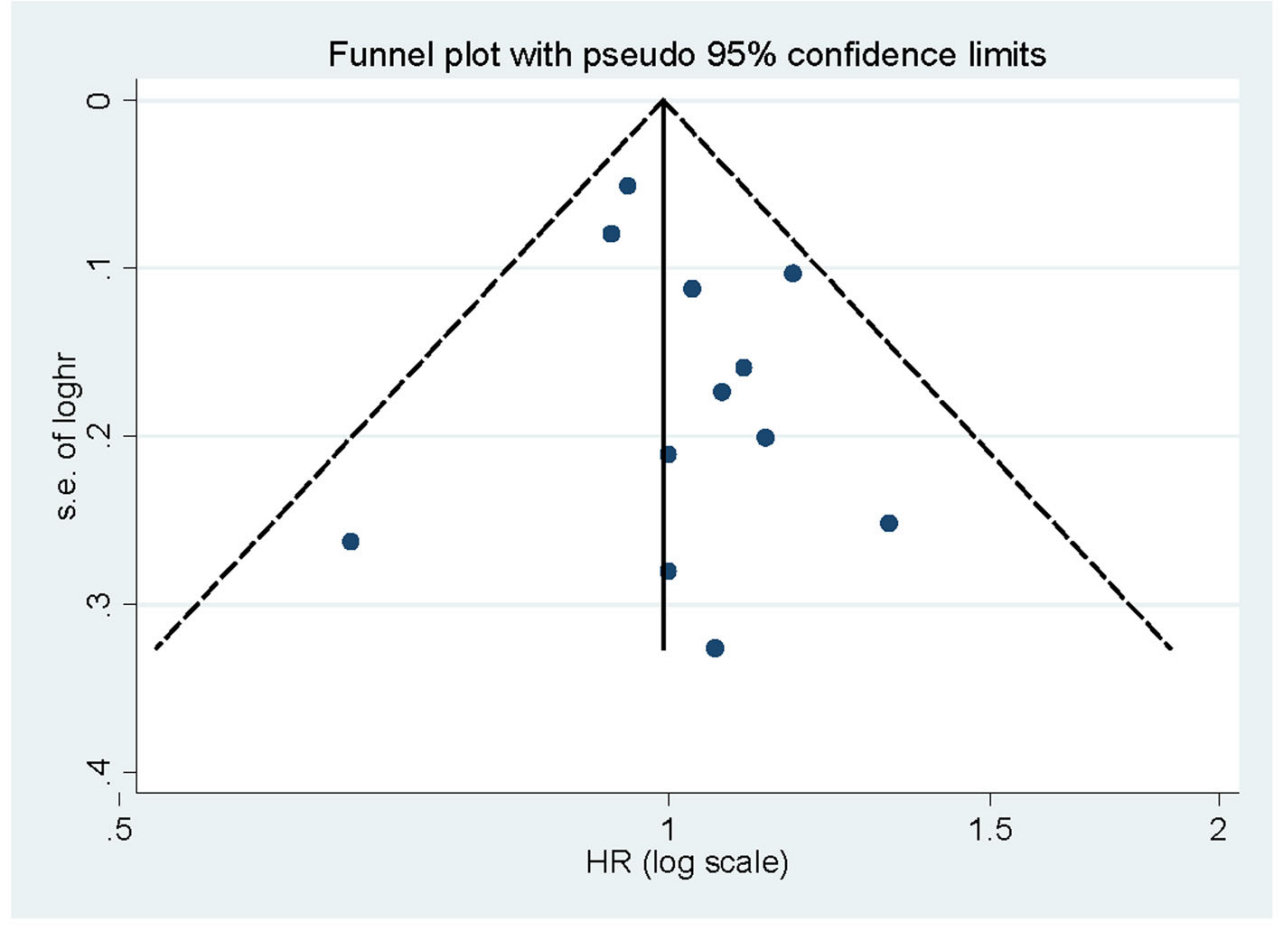
Funnel plot for assessment of publication bias

**Fig. 4:**
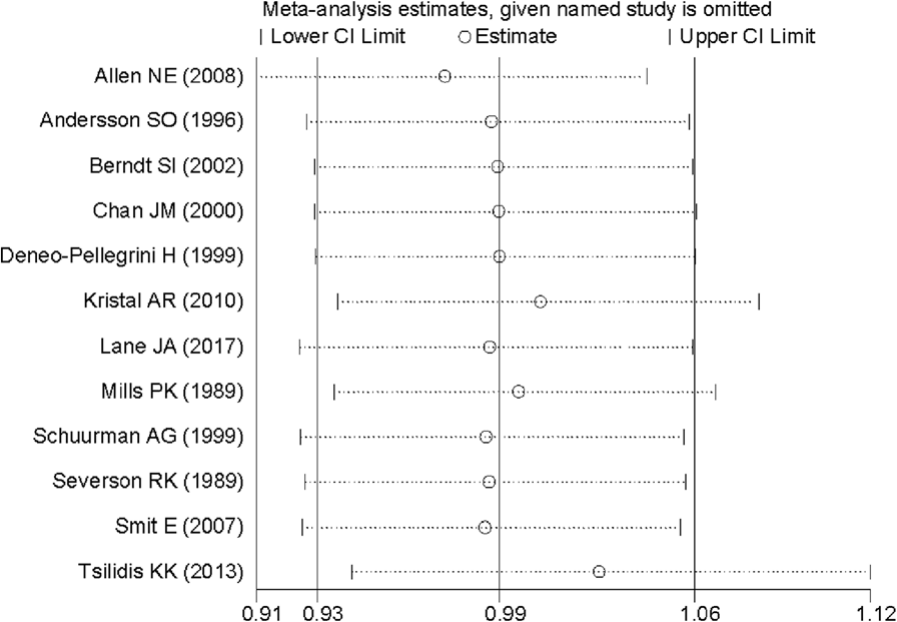
Sensitivity analysis of the association between dietary protein intake and prostate cancer risk

Begg’s funnel plots (Fig. 3) and Egger’s test (*P* = 0.296) indicated that no publication was found in overall analysis. The sensitivity analysis (Fig. 4) showed that there is no single study that had potential effects on the overall result while removing a study at a time.

## Discussion

The overall analyses suggested that high intake of protein were not related to the risk of prostate cancer. The association was significant on prostate cancer risk neither with animal protein intake nor with vegetable protein intake. We did not find any relationship between geographic locations and study design and prostate cancer risk.

Protein is an important nutrient for the human body, which is essential for body growth and development, as well as the transport of many important substances. Protein deficiency can lead to growth retardation, nutritional edema, or may even endanger life [25]. Meat is an important source of protein. A previous publication of meta-analysis was performed to explore the association on dietary meat intake for prostate cancer risk. No significant relationships were found on prostate cancer either in total red meat consumption or fresh red meat consumption, while higher processed meat consumption could increase the risk of prostate cancer [26]. In our studies, we did not obtain a positive result for prostate cancer with animal protein intake, which is consistent with the above result. Soy food is another source of protein. A study of meta-analysis suggested that soy food consumption could decrease the risk of prostate cancer [27]. The reason for this result may be that soy foods contain many fibers and phytoestrogens that block the cell cycle, induce apoptosis, and inhibit angiogenesis. Therefore, these mechanisms may support the point that higher category of dietary soy foods intake could reduce the risk of prostate cancer [25]. However, we cannot conclude a reverse relation between vegetable protein and prostate cancer risk. Furthermore, a recent meta-analysis suggested a nonsignificant association on colorectal cancer risk while in high-protein intake [28], as consistent with our results.

The most relevant risk factors on prostate cancer risk had been addressed. Previous study indicated that there was a slight association between metabolic syndrome and prostate cancer (RR = 1.17, 95%CI = 1.00–1.36, *P* = 0.04) [29]. Results from 11 cohort studies found that diabetes mellitus could significantly increase the incidence of prostate cancer [30]. However, a meta-analysis of 14 prospective studies concluded that cholesterol level in blood was not associated with the risk of prostate cancer [31].

Our study had some strength. Firstly, large numbers of cases and participants were included in this study, yielding a more comprehensive result. Secondly, no between-study heterogeneity was found either in the whole analysis or in the subgroup analyses in this study and this may obtain a stable result. Thirdly, the small study effect was not detected using Begg’s funnel plots and Egger’s test in our analysis.

To our attention, some potential limitations exited in our report. Firstly, only English or Chinese language publications were searched and all suitable studies were English articles, this may omit some other languages publications. However, no publication bias was found. Secondly, most studies included in this report were European or American populations, and we found significant association neither in European populations nor in American populations. As we know, although the results were consistent in different subgroup analyses by racial, men of African descent showed a high incidence of prostate cancer and it may make some difference [32]. Furthermore, there exists a gap in the knowledge with lower risk populations where dietary or environmental risk may play a disproportionate impact (for example, incidence of prostate cancer is lower in Asians than in Caucasians or African Americans in the USA; however, the incidence of prostate cancer in Asian Americans, while lower than that in Caucasians, is nonetheless higher than that in East Asians). Thus, more studies conducted in other countries should be performed to further assess the association on prostate cancer risk with protein intake. Thirdly, three of the included studies followed with case-control design may lead to inherent recall and selection bias of retrospective studies. Although different kinds of studies were included, we did subgroup analysis to exclude the interruption. There is no significant relation between prostate cancer and study design. Fourth, as understanding of prostate cancer growth, genetics, and the natural history of the disease has grown, subsidiary questions are increasingly important. Given the commonality of low-risk disease and evidence of over treatment, a more refined question to be asked is the association with high-grade or intermediate or high-risk disease. Therefore, more refined studies are wanted to answer these questions due to the data from an epidemiological standpoint that does not exist to support such analysis.

## Conclusions

In conclusions, findings from this meta-analysis concluded that there is no effect on prostate cancer with high-protein intake. Since some limitations exited in our study, future studies are wanted to confirm the result.

## Abbreviations

CI: Confidence intervals
RCTs: Randomized controlled trials
RR: Relative risk
